# Ethical preparedness in genomic medicine: how NHS clinical scientists navigate ethical issues

**DOI:** 10.1136/jme-2023-109692

**Published:** 2024-02-06

**Authors:** Kate Sahan, Kate Lyle, Helena Carley, Nina Hallowell, Michael J Parker, Anneke M Lucassen

**Affiliations:** 1 Ethox Centre, University of Oxford Nuffield Department of Population Health, Oxford, UK; 2 Wellcome Trust Centre for Human Genetics, Oxford, UK; 3 Clinical Ethics, Law, and Society (CELS) Oxford, Nuffield Dept of Medicine, University of Oxford, Oxford, UK; 4 South East Thames Regional Genetics Service, Guy’s & St Thomas NHS Foundation Trust, London, UK; 5 Centre for Personalised Medicine, University of Oxford, Oxford, UK

**Keywords:** Ethics- Medical

## Abstract

Much has been published about the ethical issues encountered by clinicians in genetics/genomics, but those experienced by clinical laboratory scientists are less well described. Clinical laboratory scientists now frequently face navigating ethical problems in their work, but how they should be best supported to do this is underexplored. This lack of attention is also reflected in the ethics tools available to clinical laboratory scientists such as guidance and deliberative ethics forums, developed primarily to manage issues arising within the clinic.

We explore what ethical issues are being experienced by clinical scientists, how they think such issues could be best analysed and managed, and whether their practice might be enhanced by more situated approaches to ethics deliberation and practice such as ethical preparedness. From thematic analysis of cases presented by clinical scientists at a specially convened meeting of the UK Genethics Forum, we derived three main ethical themes: (1) the redistribution of labour and responsibilities resulting from the practice of genomic medicine; (2) the interpretation and certainty of results and (3) the proposal that better standardisation and consistency of ethical approaches (for example, more guidelines and policy) could resolve some of the challenges arising.

We argue that although standardisation is important for promoting shared understandings of good (including ethical) practice, supplementary approaches to enhance and sustain ethical preparedness will be important to help clinical scientists and others in the recently expanded genetic/genomic medicine environment foster quality ethical thinking.

## Introduction

The ethical issues encountered by clinicians in genetic medicine are well rehearsed. However, the ethical challenges that arise within the clinical laboratory are less well understood.[i]

While guidance^1^ and deliberative forums^2^ have supported management of issues arising in genomic clinical practice, laboratory scientists remain undersupported due to an incomplete understanding of the range and complexity of issues they experience. This is an important concern as ethical challenges are becoming increasingly common. For example, the UK Genethics Forum, established primarily to assist clinicians with ethical and legal issues arising in genetic/genomic medicine, is now encountering an increase in ethically complex cases within the laboratory setting, or involving clinical scientists.^2^

Recent expansions in delivering genomic medicine alongside technological advances may contribute to this by increasing the complexity of clinical genetic science work in the laboratory, as well as the reach of ethical issues beyond the clinic and into different parts of the workforce. One major expansion to genomic medicine in the UK National Health Service (NHS) has been the new Genomic Medicine Service (GMS), supported by research initiatives which aim to integrate knowledge generation in genomics seamlessly within clinical care delivery.^3 4^ Accompanying this expansion are recent technological advances to genomic approaches within the Service such as the use of whole genome sequencing (WGS), in addition to targeted gene panels and single gene tests. This requires clinical scientists to fulfil requests for tests of multigene panels, particularly in rare disease and cancer genomics.^5 6^

Service expansion and technological advances such as these are changing the range and complexity of ethical issues experienced by clinical scientists in at least two ways. First, transitioning from a specialised to a mainstream service requires that clinical scientists consider ethical issues in ways that accommodate the considerations of an expanded multidisciplinary team (MDT). Second, new kinds of ethical dilemmas are arising, resulting from having to analyse much broader swathes of the genetic code than previously required, such as what information from a WGS analysis will be useful to report to clinicians and how best to present that information.^6^

We decided to use the UK Genethics Forum to explore types of ethical issues arising in laboratory practice, how these issues are being supported and managed, and suggestions for improving support and practice. First, we examine clinical cases presented by genetic/genomic clinical scientists and other genetic/genomic specialists at a meeting of Genethics. Second, we examine the proposal by some meeting attendees that increased standardisation and consistency of approach, for example, through more guidelines and policies, might adequately support their practice. Third, we suggest how the recently described concept of Ethical Preparedness might complement the use of tools such as guidelines.^7–9^

## Methods: cases from UK Genethics Forum

### Setting

Since 2001, the UK Genethics Forum has been a national case-based forum for discussion of ethical and legal issues arising in genetic/genomic medicine. The primary goal of the Forum is to support genomics professionals in ‘ensuring that ethical considerations inform the day-to-day practice of their units’^2 10^ and promote the sharing of experience and good practice in addressing ethical questions. In addition to its primary case-based function, Genethics has also played an important role in informing policy development. It is frequently judged a reliable source of advice and support, facilitating in-depth discussions regarding ethical and legal issues within cases that arise in clinical genomic practice.^1^ Healthcare professionals are encouraged to present ethically challenging cases they would like help with, which are examined within the group, and the ethical dimensions of various management approaches are explored and discussed. In what follows, we report on three such cases and the discussions which followed each of them.

### Data collection and analysis

At the November 2021 online meeting of the UK Genethics Forum, cases were presented focusing on ethical issues within laboratory practice. The meeting was organised in collaboration with the CanGene CanVar project, as well as using existing Forum mail lists.^4^ This ensured meeting attendance from a diverse range of healthcare scientists and clinicians within genetic/genomic medicine practice and research. Most attendees were from the UK, but also included healthcare professionals from other European and Middle Eastern countries. The meeting opened with a plenary talk (KL), which set the scene for the rest of the meeting by synthesising ethical issues in laboratory practice which had been brought previously to Genethics. This authorship (AL, MP, KL, HC, KS) facilitated the meeting alongside the Genethics Forum team.[ii]

The meeting was audiorecorded and transcribed. Thematic analysis was conducted to identify the ethical issues at stake within the cases. Below, we draw on some of these cases to illustrate the ethical challenges that genomic medicine raises within laboratory practice.

## Case discussions and issues

Analysis of the discussions suggested three key themes: (1) the redistribution of labour and responsibilities resulting from developments in the practice of genomic medicine; (2) the interpretation and certainty of results and (3) the pursuit of standardisation and consistency to tackle the challenges that arise in the field. The following sections provide the contextual background to each theme, a detailed illustrative case study, and a synopsis of the ensuing discussion.

### Redistributing responsibility

Navigating the new landscape within the GMS raises new challenges for clinical scientists, as they explore the boundaries of knowledge and capabilities among non-specialists, and seek effective ways to communicate for optimal outcomes. With this comes a sense of a redistribution of responsibility: scientists consider their judgements and interpretation decisions have implications for clinical practice with ensuing changes to their sense of responsibility. This set of issues is highlighted by the following example:

#### Case 1: Wilson’s disease and reporting of carrier status

Wilson’s disease is an autosomal recessive disorder of copper metabolism which, left untreated, can cause organ damage, meaning prompt identification of affected individuals is important. The case presenter reported disagreement between different laboratories which she consulted for guidance, and the referring clinician (a paediatrician) about whether to report the carrier status for two siblings (aged 7 and 15) of an affected proband. While these siblings could be reassured they were not themselves at risk of developing Wilson’s disease, they could still have an increased risk of having a child with the disorder (depending on whether their partner was also a carrier). The testing laboratory disclosed the 15-year old’s carrier status on the assumption that they had provided their own consent for testing, yet did not disclose the 7-year old’s carrier status arguing that individual could provide their own consent in the future. The fact that the reporting of the carrier status in the two siblings was being handled differently was an issue for the team.[iii] With respect to the 7-year old’s result, the testing laboratory intended to report that Wilson’s disease was excluded, but not disclose whether the child was a carrier. However, the paediatrician thought carrier status should be reported, as did the clinical geneticist, whose view was that that the possibility of a carrier status result should be raised with the family during consent for testing. This conflicted with approaches taken by other laboratories (with whom the case presenter had consulted as part of her decision-making) which would not make a carrier status finding explicit in the main body of the report, but put it further down the report such as in the laboratory analysis genotype box.

#### Group discussion of this case

Meeting attendees responded echoed the presenter’s concern that patient reports should be issued and interpreted in consistent ways, and reflected on how different referral and reporting pathways (eg, direct reporting of results to electronic health records) could change the extent to which they felt responsible for ensuring appropriate interpretation. For example, the clinical scientist quoted below described how the involvement of non-genetic/genomic clinicians would change their confidence in reporting carrier status:

at the moment we do treat mainstream clinicians differently to clinical genetic services. If we got a referral from [a] GC [genetic counsellor] and it was for a seven year-old and they said [please report] carrier status, we wouldn’t hesitate to report the carrier status back because we would assume that they’ve been properly consented. But we wouldn’t do that for mainstreaming. (l1343-7)

Attendees questioned how much interpretation work non-specialist clinicians and patients could be expected to do for themselves, one attendee noting that demands on paediatric services might mean there is still a risk that the family in Case 1 would not be adequately counselled or followed up about the clinical significance of a carrier test result (l1553–7). This highlights a concern about where responsibility should reside: who should be responsible for following up families or individuals with genetic results, especially when mainstream services are not set up to follow up future risks. Importantly, discussions indicated that discharging this responsibility was more complicated than genetic/genomic specialists making written reports or letters more detailed or explicit, one attendee noting how clinical consultation in the mainstream setting can also change the meaning of written information:

we [genetic specialists] have all of the skills to, and the knowledge to counsel these patients, but… [thinking about] the mainstream clinicians, sometimes even if you document it in your letter, at the time you feel that they’re well understood, but the message received or perhaps repeated to a family member or another clinician is not the precise genetic counselling language that we would use, and that message can get changed when it’s repeated. (l1775–80)

### Interpretation and certainty of results

As genetic testing possibilities have expanded (from primarily single gene tests to also whole genome approaches) clinical scientists experience more challenges related to the uncertainty of test results, including dilemmas about what findings ought to count as ‘a result’ that should be in the patient health records versus more exploratory and research-oriented findings. These dilemmas are reflected in the literature on new variant classification frameworks which seek to improve classification of variants of uncertain significance (VUSs), but which nevertheless recognise the need to strike a balance between ‘the clinical benefit of [moving] classification of a variant out of the “VUS” category against the harms of erroneous misclassification’.^11^

This set of issues was highlighted in the following case from outside the UK about whether to use preimplantation genetic testing for monogenic disorders (PGT-M; previously known as preimplantation genetic diagnosis, or PGD] to test for VUSs.

#### Case 2: PGT-M using a VUS result for reproductive decision-making

The case concerned a consanguineous couple whose first child had global developmental delay and multiple congenital anomalies and had died in early childhood.

The child had been found to have two variants of uncertain significance in genes which were potentially in keeping with the child’s phenotype. One was a de novo heterozygous variant associated with an autosomal dominant condition, the other was a homozygous variant (inherited from each parent) associated with an autosomal recessive condition. The couple wished to have another child through preimplantation genetic testing; however, this was declined by the state healthcare laboratory/clinic due to the uncertain nature of the variants identified. A commercial lab had agreed to undertake PGT-M for these VUS, on the basis that their exclusion might be of some value. However, this testing resulted in the couple losing the ‘uncertain’ meaning from the VUS term—they were given the impression that this testing was significant and would ‘avoid [them] having another affected child’.

The state healthcare laboratory/clinic which held a sample of DNA from the deceased child refused to release the sample to the parents to be used in PGT-M by the commercial laboratory on grounds that there was no clinical utility for VUS testing. Discussions highlighted that the motive for withholding access to the sample was seen as obstructive by the parents.

#### Group discussion of this case

Ethical issues in this case centre on suggestions to use a VUS to exclude putative diagnoses in future offspring. Meeting attendees discussed the caution needed in being tempted to ‘turn data into information prematurely’. This reflects the judgement needed when deciding whether to maintain an approach consistent with current guidelines to conventionally less actionable categories of genomic data for example, VUSs in the clinic, or to derive more clinically significant or actionable findings where the clinical context or phenotype is driving this.

While these developments in technology have been helpful reducing the length of the ‘diagnostic odyssey’ there was concern about genomics being seen as providing clinical answers it often could not provide. Some raised concerns that genetic/genomic technologies might lead to a new type of ‘odyssey’. Later in the discussion, another attendee described this as a tension between the desire for genomic results to be clinically useful yet being cautious of the impact of misinterpretation:

there’s this kind of to and fro, like you really want to find an answer for your patient, but you don’t want to give them the wrong answer. But because you really want to give an answer to your patient, you kind of are slightly more invested in [interpreting] these slightly woolly variants [as possibly significant] than the lab are. (l1006–9)

This highlights the importance of context and clinical presentation when interpreting genomic results and therefore how the perspective and responsibility of those generating and interpreting these results is a significant ethical factor.

### Consistency and standardisation

The purpose of professional standards is to promote shared understandings of good (including ethical) practice, especially in rapidly evolving areas of healthcare. Standards are developed and promoted via a number of methods in genetic/genomic medicine, for example, the need to meet competences during training,^12^ professional guidelines^1 13 14^, consensus standards such as from the Cancer Variant Interpretation Group UK,^11^ and local policies (to take the Case 3 example a Genomic Laboratory Hub (GLH) might create standards for offering extended cystic fibrosis (CF) testing). However, while working to standards through written materials and training is valuable, standardisation alone is insufficient to support ethical decision-making when considering the extent and range of ethical issues currently being experienced by practitioners.

This issue is illustrated by a case suggesting guidelines and training to resolve different management approaches to the finding of echogenic bowel on fetal ultrasounds.

#### Case 3: echogenic bowel finding used for extended carrier testing for CF

A geneticist presented two cases in two different pregnancies with different practices on finding echogenic bowel at the 20-week anomaly scan.^15^ [iv]

In the first case, parental testing revealed the mother was a carrier for a variant in the CF gene, which was also detected by amniocentesis in the fetus. The father did not carry a common CF variant but as rare variants had not been excluded, there was still a residual risk that the fetus might have CF. While the clinical geneticist wanted to offer extended CF carrier testing to the father, the laboratory commissioned with doing the test thought this would be a poor use of resources. However, another laboratory then agreed to offer this extended testing. In a second case, where again, only one parent was found to be a carrier following testing of common CF variants, more extensive testing was available but the obstetrician decided not to pass this offer on to the parent, possibly as a result of their experience in the first case.

These two cases raised a number of ethical issues. The team was uncertain about whether to offer extended CF carrier testing because of a low chance of finding carrier status through extended testing and scarce resources. Second, it was not clear how to interpret a VUS from extended testing and, whether CF prediction was clear enough to offer late termination on the grounds of fetal disability.[v] The case presenter then raised the issue of varying degrees of consensus between different clinical genetics centres and laboratories with respect to offering extended testing. She suggested that variation was perhaps becoming more apparent since the reorganisation of labs into different GLHs. The presenter argued that this variation could lead to systemic inequity in patient treatment and wanted to ensure “that we’ve got a very clear sort of protocol and policy within our department and with the lab.” (l1903–5)

#### Group discussion of this case

In discussions, the solution of consistency and standardisation (usually by producing written materials like guidelines) was proposed to address the ethical issues arising. For example, the presenter of Case 1 (a clinical laboratory scientist) proposed the following measures:

Really, really important that we need consistency across the labs [about routinely reporting carrier status] because even within our GLH we were doing different things, so it’s really important that we have guidelines and then we agree what we’re doing. (l1351–3)

This was echoed by other calls for protocols and ‘unambiguous policy for labs to be able to standardise across the UK’, and for ‘agreed principles [to be] applied uniformly’ across mainstream, clinical genetics and the spectrum of genetic testing (l1097ff).

## Discussion

In this study, we have presented cases and ensuing case discussions from a UK Genethics Forum meeting, which brought together genetics professionals from a range of different settings. The cases and discussions presented at the meeting and reported here demonstrate how some of the ethical challenges arising for genetic/genomic clinical scientists are linked to developments in genomic techniques and approaches, meaning that more variants are discovered whose clinical significance may evolve, emerge or remain uncertain. All this is underpinned by ongoing efforts to make structural changes within the NHS. We have also noted the tendency for case presenters and meeting attendees to call for standardisation of practice through written materials such as guidance and training. However, there are reasons to think that standardisation might not by itself be sufficient to resolve the issues being experienced.

First, leaving aside the importance of treating patients equitably,[vi] it seems generally important to recognise that disagreements can be productive and can justifiably improve practice. For example, people might reasonably disagree about the course of action to take as part of a justified process which works through these disagreements.^16^ This suggest that standardisation needs to allow space for reasonable disagreement. The call for standardisation should not be taken to mean that disagreements do not have an important role in practice. Second, looking to professional standards alone to resolve practice dilemmas will be counterproductive. This is because standards will not dictate an approach but rather will suggest a range of possible actions to take, each requiring interpretation in particular cases. Also standards will change over time through interpretation and usage. Third, relying on production of more standards from the genetic/genomic specialism might send mixed signals about the extent to which the specialism (and actors within it like clinical scientists) should lead on responsibility for ethical practice within the GMS. On one hand, specialists will still count as leaders for formulating quality approaches to practice since they are more expert and up to date with new approaches and their ethical ramifications, for example, WGS. On the other, the reality of mainstreaming genetics/genomics means it is practically necessary and valuable that responsibility for decisions about ethics and practice should be more widely distributed among specialists and non-specialists (including patients). In this way, the ethics of the Service and specialism can benefit from a distributed, deliberative practice environment.

It is here that the concept of ethical preparedness (EP) might help supplement existing resources such as guidelines or methods of standardisation.

### The concept of EP

The concept of preparedness has been used in relation to emergencies in public and global health.^7 17 18^ Preparedness describes how, when there is a public health emergency, agencies, systems and structures should be properly prepared to manage it.^19^ The concept of EP has been used in public health,^20–22^ research^8 23^ and genomics^7^ to describe a capability, opportunity and motivation to respond to the ethical issues arising in a particular clinical situation, as well as being able to anticipate ethical concerns in advance in areas where practice is rapidly evolving due to, for example, crises or technological advances. This is on the basis that just as emergencies may be unpredictable and evolving, so too researchers and health professionals should be prepared to ‘face new challenges born of the complexity, uncertainty and longevity of technologies’.^23^ The concept encourages those involved in particular situation to be prepared to consider ethical issues *in situ* and appreciate that particular nuances will often not be answered by off the shelf solutions from guidelines or legislation. EP has been described as follows:

‘ethical preparedness [is] a state from which one is able to identify and articulate ethical issues in a timely and ongoing manner, and where (ideally) one has the tools and the skills/experience available to address them. In the absence of the latter, one must know whom to consult and engage with in order to avail of appropriate expertise’ e.g. ‘professional bodies, clinical ethics committees, regulators and indeed patient groups’.^7^

Practical implementation of EP in genetics/genomics might involve organisations like the NHS prioritising the time and space for quality ethical MDT discussions and deliberations (as well as access to expert groups when needed), such that they become part of day-to-day practice across the genetic/genomic specialism. In that way, the approach to ethical reflection is flexible and responsive to evolving practice. This affords professionals the opportunity to cultivate the ethical tools and experience to address issues within the practice space as they arise.

### How ethical preparedness might support professionals in managing practice in this environment

First, employing EP can make use of and contribute to an ethically reflexive practice environment. This is because its model is to embed ethical thinking within the practice environment, so using the environment (and the range of views therein) to inform practice judgments. Second, implementing EP requires a sensitivity to context, not just compliance with standards, policies or guidelines. This allows practitioners to make decisions with reference both to guidelines and also to context. Because contexts will evolve alongside the delivery of genomic medicine, this also gives potential for similar cases, for example, Case 1’s carrier disclosure cases, to result in different decisions as modulated by context. Contextual sensitivity also alleviates concerns about inconsistent practices since it yields reasons which may justifiably lead to different outcomes. However, using professional standards and genetic/genomic specialist advice is still important for ensuring decision-making is of sufficient quality. Practitioners should be able to call on standards, guidelines or similar resources to ground their decisions, using these as a knowledge base and a touchstone for accessing a genetics/genomics community of practice. Thus in order to support practitioners in moving to an EP approach, it is important to recognise that making an ethical decision should not rest wholly on their moral character or competencies.^8^

Using an EP approach should also not discount existing, more formal opportunities for ethical practical deliberations such as the UK Genethics Forum and MDTs.^24^ These also provide opportunities for practitioners to develop skills in ethical deliberation, and provide a different way to encounter and learn from diverse viewpoints.^1 2 25 26^ Properly situated EP would involve organisations arranging the time and space for quality ethical MDT discussions and deliberations such that they become part of professional structures and culture, and are routine in everyday clinical practice.^8^

## Limitations

Genethics Forum meetings bring together a diverse range of genetics professionals at different careers stages, from different types of settings, and from different parts of the country, generating a diverse range of perspectives. Inevitably, however, this is limited to those who do in fact attend such meetings. A greater number of meetings might have generated more themes. Yet by specifically targeting the meeting call towards issues experienced by clinical scientists, we developed out of the meeting a rich case study for exploring our research questions, and how proposed ways of managing issues arising via compliance-focused approaches might be insufficient.

## Conclusion

Genetic/genomic medicine continues to present complex ethical issues to all areas of service provision. We have highlighted one area— the clinical science laboratory—which is currently experiencing issues of increasing complexity. In exploring the experiences of clinical scientists presenting cases to the UK Genethics Forum, we have revealed three main pressing concerns. First, that clinical scientists feel partly responsible for how patients will receive results and how they should be followed up in future. Second, that testing using increased panel sizes may cause scientists (or others delivering GMSs) to feel obliged to turn data into clinically significant findings prematurely, in order to ‘give an answer’ to patients. Third, that being consistent in the approach to similar clinical presentations is important to treat patients equitably, but is not a complete solution to managing the range of ethical issues being experienced. In that sense, resources to promote standardisation (eg, more guidance and policies) represent only part of the solution to the question of managing and supporting clinical scientists in their ethical practice.

In addition to such resources, we recommend a more wide-reaching, fundamental intervention of EP. This situated approach to ethics deliberation uses the context and diversity of the practice environment to inform quality ethical thinking, and suits the expanding and rapidly transforming nature of genomic medicine delivery. The EP approach can be combined with existing ethics tools such as guidance and professional standards to maintain quality ethical thinking, and it is also consistent with more formal opportunities for ethics deliberation such as the UK Genethics Forum.

Implementing EP involves prioritising quality ethical MDT discussions and deliberations, such that they become part of regular practice in genetics/genomics (and other specialisms in future). In practice, this might involve optimising the settings for local ethics discussions, providing access to clinical ethics committees and to formal structured resources such as the UK Genethics Forum and online genethics resources. However, these resources should also aim—as part of their remit—to better empower healthcare professionals to recognise and value their own abilities in managing ethical issues over time.

## Footnotes

Clinical laboratory professionals include both genetic technologists who generate data from biological samples—so called ‘wet’ lab work—and clinical scientists or healthcare scientists who interpret genomic data—so called ‘dry’ lab work.

Please see the Acknowledgements section for team details.

Professional guidelines pull in different directions in such cases, both suggesting results should be reported at different times, and suggesting that both sets of results might reasonably be given to parents for their communication. See Consent and confidentiality in genomic medicine guidance in reference list which discusses a similar case.

Echogenic bowel is a finding on antenatal ultrasound where the bowel appears brighter than expected and is ‘a “soft marker” [meaning it holds only weak predictive value]… for a variety of conditions including chromosomal disorders, intrapartum infections, antepartum events and cystic fibrosis’ or CF.

Termination of pregnancy beyond 24 weeks is permissible in the UK when a fetus is expected to have a ‘severe handicap’.

As in Case 3, equitable treatment of patients would be an area where standardisation could be crucial, noting also better outcomes on equity are a key aim of the GMS.
